# Sport training of axial rotation affects spatial ability: Evidence from behavior and fNIRS

**DOI:** 10.1016/j.heliyon.2024.e39340

**Published:** 2024-10-13

**Authors:** Tian Feng, Fuchun Zhang, Manqi Liang, Jinzhao Liu, Youxin Wei

**Affiliations:** a School of Physical Education, Henan University, Kaifeng, Henan, China; b Department of Sports, Henan Sport University, Zhengzhou, Henan, China; c School of Physical Education, Zhengzhou University, Zhengzhou, Henan, China

**Keywords:** Mental rotation, Axial experience, fNIRS, Frontoparietal area, Parieto-occipital area

## Abstract

The present study investigated the spatial ability of adolescent athletes with different sport expertise of axial rotation, providing the cognitive characteristics from both behavior and functional near-infrared spectroscopy (fNIRS) tests. 16 basketball and soccer players, 15 freestyle athletes and 15 runners were selected for spatial ability testing with a three-factor mixed experimental design of 3 (sport type: open high-spatial sport, closed high-spatial sport, closed low-spatial sport) × 2 (rotation angle: 45°, 90°) × 3 (rotation axis: horizontal, vertical, longitudinal). Repeated-measures ANOVA was performed to evaluate the behavioral and fNIRS data of the mental rotation test. The behavioral data showed that the closed high-spatial (CH) group showed better performance than the open high-spatial (OH) and closed low-spatial (CL) group for large angle for every rotation axes. The fNIRS results showed the CH group had greater brain activation than the OH and CL group in the left dorsolateral superior frontal gyrus, precentral gyrus, postcentral gyrus, superior parietal gyrus, left angular gyrus, left precuneus and middle occipital gyrus. The findings provide additional empirical support for relating body rotation experience to spatial cognition performance.

## 1 Introduction

Spatial ability is a general expression of intelligence in the spatial cognitive system and refers to the ability to generate, retain, extract, and transform visual images [1]. Spatial ability is a basic ability necessary in daily life, whether in daily social life or in the process of learning and completing sports movement skills. All people need good spatial ability to complete precise and complex daily tasks or sports activities. Brain science research has shown that the spatial cognitive advantage of elite athletes is demonstrated by faster and more efficient cognitive processing in the brain [2].

Mental rotation (MR) ability is one of the important components of spatial ability and is an individual's ability to hold and manipulate or turn objects in space. Researchers have conducted numerous studies and reported many measurement schemes that can measure individuals' mental rotation ability more objectively and quantitatively [3]. The classical mental rotation test was designed by Shepard and Metzler [4]. As most researchers have shown [4,5], RTs increase gradually as a function of angular disparity, which means that the more the angle is rotated, the longer it takes the participants to react. This may account for the cognitive load at different angles. The cognitive load is one of the factors that influences mental rotation ability [6]. Studies have confirmed that as interference (i.e., large angular disparity) intensifies, participants' processing of information increases [7,8].

As a type of representational operation, mental rotation involves similar cognitive processing to that of real rotation [9]. A previous study revealed that the motor areas of the brain are also activated when individuals mentally rotate [10]. Therefore, mental rotation ability is closely related to motor expertise [11]. Some studies have compared the mental rotation ability of individuals with different degrees of sports experience and reported that the mental rotation ability of expert athletes is better than that of ordinary people [[12], [13], [14]]. Athletes manipulate and move their bodies in space to practice and improve their skills. Therefore, the advantage for athletes in mental rotation ability after years of training is a process of embodiment. In light of embodied cognition, cognitive processing is based on the physical state [15,16], and all types of cognitive activities, such as thinking, classification and judgment, are not just information processing in the brain but are closely related to one's physical properties, perception, and movement ability.

Previous studies have shown that the embodied MR ability of athletes is moderated by sport type. Studies have shown that gymnastics and orientation movement player have better mental rotation abilities than soccer and running participants do [13,17]. Accordingly, it has been further suggested that the stronger the correlation between mental rotation task stimuli (direction, angle and axis et al.) and sports experience is, the better the athletes' performance and that the mental rotation ability of athletes in different sports shows “selective influence” [18]. According to the perspective of action imitation theory, some mental representations and internal mechanisms are shared between motor representations and action execution [19]. After training, athletes’ nervous system can coordinate with sensory input, thus accelerating the perception of their surroundings and increasing the efficiency of the use of psychological resources [20]. However, some studies concerning the spatial essentials of events fail to elucidate the differences between high-spatial sport and low-spatial sport. Sylvie and Larue used two-dimensional cube MRTs to compare sports with high rotation requirements (gymnastics) and low rotation requirements (handball, rugby, basketball, football, badminton, wrestling, judo, track and field) [21]. Habacha and Molinaro investigated the MR performance of spatial (football, handball, basketball, racket, hockey, gymnastics) and non-spatial (track, wrestling, and swimming) players in three-dimensional MRTs [22]. Moreover, Sylvie and Jacques examined the MR ability of open sports (rugby, basketball, soccer, badminton, wrestling, judo and tennis) and closed sports (medium distance running, cycling, swimming, gymnastics, archery, javelin, and fitness) [23]. According to previous research, closed sports include a stable environment and predictable events (i.e., swimming), but open sports require fast and frequent reactions to unpredictable environmental changes (i.e., basketball) [23]. However, these methods did not distinguish among different spatial sport types, indicating that the classification method may need to be modified.

Moreover, stimulus type (i.e., rotation side, direction and angle) can strongly influence the performance of motor experts [24,25]. Researchers reported that table tennis players made faster judgments on the dominant hand than on the nondominant hand in a mental rotation task [24]. The reactions of gymnasts were faster than those of nonathletes only in their dominant rotation direction, and they found that soccer and handball players performed better on the task of vertical axis rotation than on the task of horizontal rotation [25]. Divers were faster than nonathletes were in judging images at an inverted angle of 180° and showed differences in brain activity [26]. Thus, axis is a vital index of rotational movements. Using the spatial coordinate system as a reference, the axes of human rotation can be divided into the left‒right horizontal axis, the up‒down vertical axis, and the front‒back longitudinal axis. Fargier et al. compared the MR performance of futsal and rhythmic gymnasts and sedentary people and reported that the reaction time of the futsal group was shorter in the frontal, horizontal, and sagittal planes than that of the sedentary group, and the reaction time of the futsal group in the horizontal plane was also shorter than that of the rhythmic gymnastics [27]. However, the question of whether athletes skilled in multiple-axis body rotation, like aerial freestyle skiers, have superior MR abilities compared to those who typically rotate around a single axis is still not clear.

Studies addressing the age of athletes have shown that there is one or more sensitive periods for adolescent cognitive development [28] and that spatial ability has a linear effect with age [29]. It has also been found that students' spatial ability is weak until the age of 10, students' spatial ability develops rapidly after the age of 10 and increases with age, and spatial ability tends to mature around the age of 14–17 [[30], [31], [32]], which means that the adolescent stage is a sensitive period for the development of spatial ability; therefore, adolescent athletes were chosen as the subject of this study.

The use of functional near-infrared spectroscopy (fNIRS) is rapidly increasing in the field of cognitive neuroscience due to its near-harmless and portable nature. fNIRS has a wide range of requirements for experimental settings, physical movements, and the population that can participate, especially for outdoor experimental settings, more strenuous movements, and newborns and elderly individuals, respectively [33]. Therefore, NIR spectroscopy plays a major role in the study of motor cognitive neurology. It has been shown that the frontoparietal neural network is the main functional brain region that regulates cognitive abilities [34]. Kosslyn et al. reported that participants' mental rotation activated the precentral gyrus, superior parietal cortex, inferior parietal cortex, primary visual cortex, insula, and frontal regions [35]. There is a lack of research on brain regions related to the activation of athletes' mental rotation ability, so the frontal, parietal, and occipital regions were selected as monitoring areas in the present study to investigate the activation levels of brain regions in athletes as they performed mental rotation tasks.

In summary, to investigate the effect of motor expertise type on MR, the spatial factor of sports training, which is coupled with the characteristics of the task stimuli, especially the material angle and axis, was examined with open-closed types of sports. Therefore, the present study classifies the spatial sport type via the matrix of high-spatial/low-spatial and open/closed types. High-spatial sports are events that involve mental and physical rotations in their practice (i.e., gymnastics), and low-spatial sports refer to activities that require very little motor rotation (i.e., soccer) [21]. The present study divided three kinds of sport: (1) open high-spatial (OH) sport, such as basketball and soccer, (2) closed high-spatial (CH) sport, such as freestyle skiing and (3) closed low-spatial (CL) sport, such as running. Because open sports often involve complex spatial transformation, open low-spatial sports are not defined. A computerized MR experiment was conducted to test the influence of axial rotation experience on spatial ability. We hypothesized that: (1) the rotation angle affects the performance of MR in every sport type. (2) the OH and CH group have superior behavior performance than the CL group and that their advantages exist in certain axes or angles congruent with their training experience. (3) the CH group have lower activation in the frontal, parietal and occipital brain regions than the other two groups in the fNIRS data.

## 2 Materials & methods

### 2.1 Participants

The respective a priori sample size calculations (α = 0.05; power [1-β] = 0.95; G∗power 3.1.9) suggested a sample size of n = 30 for the smallest of these effect sizes. Therefore, forty-six adolescent players were enrolled in the present study. They were divided into three sport types on the basis of the type of sport: (1) an OH group consisting of 16 basketball and soccer players (8 males and 8 females) who were 13.31 ± 0.79 years old and had 2.23 ± 1.43 training years.; (2) a CH group consisting of 15 freestyle skiing players (8 males and 7 females) who were 14.60 ± 1.02 years old and had 3 ± 0.34 training years; and (3) a CL group consisting of 15 runners (9 males and 6 females) who were 14.60 ± 1.78 years old and had a training time of 1.27 ± 0.44 years. This study was approved by the Ethics Committee of the Physical Education College of Zhengzhou University (202202), and written informed consent was obtained from the participants and their parents prior to participation.

### 2.2 Stimuli and task

**Fig. 1:**
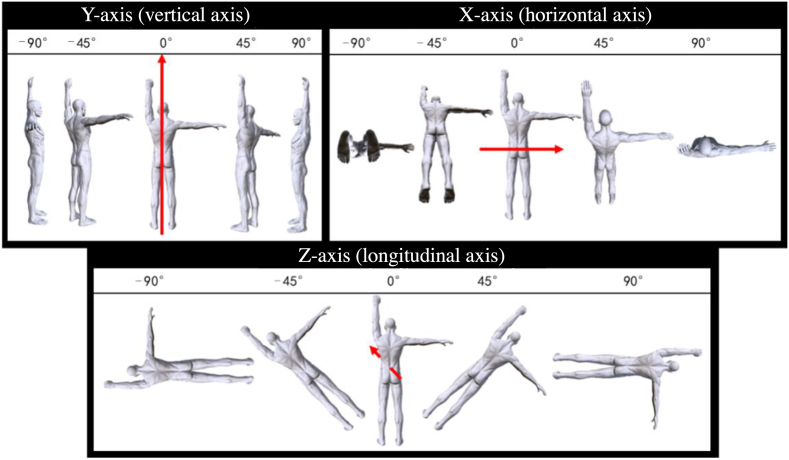
Experimental stimuli.

The experimental stimulus used was a simple body movement graphic that included a picture of a male with one arm rising upward and the other arm rising sideways. One figure was presented at a time, and the figure containing the experimental stimulus was categorized into three rotation types based on the axial characteristics of the movement. The rotation angles for each axis were 45 and 90 (Fig. 1 below). The size of each image was 14 cm × 18 cm. Stimulus pictures were presented on the screen with viewing distance about 50 cm and visual angle about 2.5° in height. The experimental task was designed by E-Prime 3.0 and presented using a 23.8-inch monitor.

### 2.3 Procedure

**Fig. 2:**
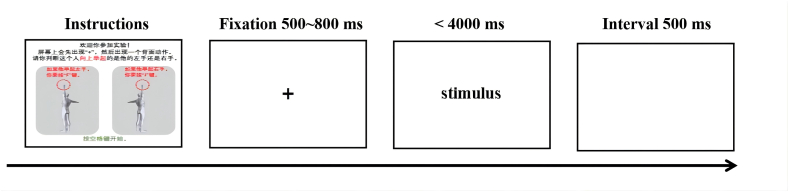
Mental rotation test task design.

The overall duration of the experiment was approximately 20 min. The subjects were required to determine in the shortest amount of time whether the mannequin in the picture was holding up the left hand or the right hand; participants were asked to press the “F" key to indicate the left hand or the “J" key to indicate the right hand. The response time and accuracy of the spatial perception task were obtained. The subjects were required to press the keys accurately and quickly without receiving any cues or feedback (positive/negative) during the experiment (see Figure 2). One block consisted of 48 trials, and after completing one block of stimuli, each subject rested for 15 s before moving on to the next block until all 6 blocks were completed; the test ended with the recording of 20 s of resting data for each subject. During this time, the subjects' behavioral and fNIRS data for the task were recorded in a file, which was circulated until the task was completed.

**Fig. 3:**
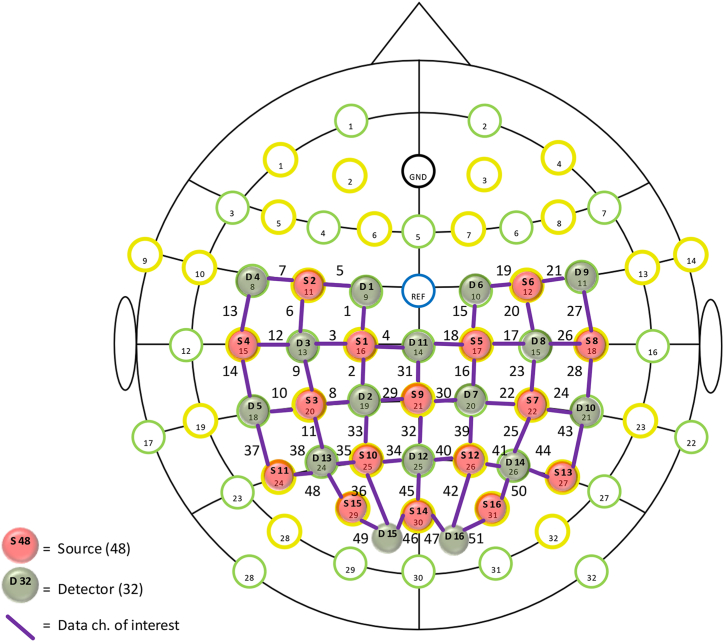
fNIRS device channel layout. Note: Source indicates the light source probe, detector indicates the receiving probe, and channel indicates the channel.

### 2.4 fNIRS data collection

In this study, an elastic head cap was used to fix the template and the head. When the detector probe and fNIRS light source probe were placed in the template, it was necessary to ensure that there was contact between the probe and the scalp, and that the subject's hair was sufficiently ruffled. The experimental probes were set to monitor the frontal, parietal, occipital, and cuneiform regions of the cortex. A total of 16 emitters (780 nm and 830 nm wavelengths) and 16 detectors, together constituting 51 measurement channels, were used to align the multichannel NIR data space to the Montreal Neurological Institute (MNI) space (see Figure 3). In terms of templates, the detection areas were divided into left frontal, right frontal, left parietal, right parietal, and occipital regions according to the ALL partition, as shown in Table 1. First, the Lambert‒Beer law was applied to the collected optical data using the NirsLab to obtain the relative change in oxygenated hemoglobin (Oxy-Hb) concentration. The analysis of cerebral blood oxygen data in this study focused on OxyHb, which is used to reflect the level of neural activation in the brain with a better signal-to-noise ratio and is more sensitive to the task response than Deoxyhb [36]. Finally, the obtained data were grouped and imported into SPSS for further analysis.

**Table 1:** Brain region and Channel of the study.

Brain region	Channel
Left frontal area	ch1, ch3, ch4, ch5, ch6, ch7
Right frontal area	ch15, ch17, ch18, ch19, ch20, ch21, ch31
Left parietal area	ch2, ch8, ch9, ch10, ch11, ch12, ch13, ch14, ch16, ch29, ch32, ch33, ch34, ch35, ch36, ch37, ch38, ch45
Right parietal lobe area	ch22, ch23, ch24, ch25, ch26, ch27, ch28 ch30, ch39, ch40, ch41. ch42 ch43, ch44, ch47
Occipital region	ch46, ch48, ch49, ch50, ch51

### 2.5 Data analysis

The data processing part consisted of two parts: behavior and fNIRS. These two kinds of data for every sport type obtained a normal distribution (z<1.966, p>0.154, in all instances). For the behavioral data, a three-factor repeated-measures ANOVA was used with the between-subject factor of sport type (OH, CH, CL) and the within-subject factors of the rotational axis (horizontal, vertical, longitudinal) and angle (45°, 90°) to analyze the accuracy and reaction time (RT) of the MR task. For the fNIRS data, a four-factor repeated-measures ANOVA for sport type × rotation axis × rotation angle × channel was conducted. For the ANOVA, the partial eta squared (η_p_^2^) was calculated as the effect size. A post hoc analysis was conducted when the interaction was significant, and the Bonferroni correction method was used for correction. The alpha level was set to 0.05 for all the statistical analyses. In the case of a violated sphericity assumption, the respective degrees of freedom were corrected according to the Greenhouse‒Geisser correction.

## 3 Results

### 3.1 Behavioral results

ANOVA of RT revealed significant main effects of rotation axis, F (2, 79) = 15.355, p < 0.001, η^2^ = 0.280, angle, F (1, 80) = 88.691, p < 0.001, η^2^ = 0.526, and sport type, F (2, 80) = 14.129, p < 0.001, η^2^ = 0.261. The interaction effects were significant for axis and sport type, F (4, 160) = 4.029, p = 0.027 η^2^ = 0.092, axis and angle, F (2, 79) = 55.525, p < 0.001, η^2^ = 0.584, but not for sport type and angle, F (2, 80) = 2.348, p = 0.102, η^2^ = 0.055. A marginally significant three-factor interaction effect was found, F (4, 160) = 2.299, p = 0.061, η^2^ = 0.054. Post hoc analyses of main effects revealed that the CH group had significantly shorter RT than the OH and CL group, the RT at small angle (747.69 ± 194.53 ms) was significantly shorter than that at large angle (509.54 ± 124.07 ms, p < 0.001), and the RT for horizontal and longitudinal rotation were significantly shorter than for the vertical rotation. Post hoc tests showed that the CH group responded faster than the OH group when mentally rotating 90° images around horizontal, vertical and longitudinal axis, and they were also faster than the CL group in vertical 45° and 90° condition. However, the CL group had shorter RT than the OH group in the in vertical 90° condition. Concerning the comparison of RTs for three axes, the results demonstrated that the OH group showed faster 90° RT for the longitudinal rotation than that for the horizontal and vertical rotation. The CH group showed faster 90° RT for the longitudinal rotation than that for the vertical rotation. The CL group showed faster 45° RT for the horizontal rotation than that for the vertical and longitudinal rotation but had faster 90° RT for the horizontal and longitudinal rotation than that for the vertical rotation (Table 2).

**Table 2:** Reaction time of MRT for every sport type, axis and angle (ms).

Sport type	OH		CH		CL		Total	
Angle	Axis							
	45°	90°	45°	90°	45°	90°		Comparison results
Horizontal (H)	552.64± 214.98	874.79± 251.32	450.44± 79.35	621.80± 84.47	436.14± 56.96	746.75± 204.64	613.76± 214.05	CH90<OH90^a^
Vertical (V)	480.21± 84.87	998.33± 199.35	452.69± 64.63	688.63± 124.59	576.04± 131.67	826.39± 186.48	670.38± 231.35	CH45<CL45^c^ CH90<OH90^a^ CH90<CL90^c^ CL90<OH90^b^
Longitudinal (L)	602.89± 143.33	714.38± 109.18	514.22± 56.80	593.49± 75.54	520.58± 79.54	664.67± 145.08	601.69± 130.06	CH90<OH90^c^
Total	719.85 ± 200.46		553.54 ± 130.40		612.447 ± 169.95			CH90<OH90^a^ CH90<CL90^b^
Comparison results		L < H^a^ L < V^a^		L < V^b^	H < V^c^ H < L^c^	H < V^c^ L < V^a^	H < V^b^ L < V^a^	

a p < 0.001.

b p < 0.01.

c p < 0.05.

The accuracy analysis revealed significant main effects of rotation axis, F (2, 75) = 11.488, p < 0.001, η^2^ = 0.235, and angle, F (1, 76) = 24.546, p < 0.001, η^2^ = 0.244, but not for the sport type, F (1, 76) = 2.484, p = 0.090, η^2^ = 0.061. The interaction of rotation axis and angle (F (2, 75) = 18.491, p < 0.001, η^2^ = 0.330), rotation axis and sport type (F (2, 52) = 2.531, p = 0.043, η^2^ = 0.074, and angle and sport type, F (2, 76) = 3.026, p = 0.054, η^2^ = 0.016, were at least marginally significant. The three-way interaction of rotation axis, angle and sport type was significant, F (4, 152) = 2.433, p = 0.05, η^2^ = 0.060. Post hoc analyses comparing the two rotation angles revealed that longitudinal axes were significantly greater than horizontal and vertical axes, and small angles (0.986 ± 0.02) were significantly greater than large angles (0.960 ± 0.05, p < 0.001).

Moreover, post hoc tests showed that the CH group showed higher accuracy than the OH and CL group when mentally rotating 90° images around horizontal axis. The CL group's accuracy for horizontal and vertical rotation at small angle was higher than that at large angle, and the OH group's accuracy for horizontal rotation at small angle was higher than that at large angle. The results demonstrated that the OH group responded higher 90° accuracy for the longitudinal rotation than that for the horizontal rotation, and the CH group responded higher 90° accuracy for the horizontal and longitudinal rotation than that for the vertical rotation. In respect for the CL group, they responded higher 90° accuracy for the longitudinal rotation than that for the horizontal and vertical rotation (Table 3).

**Table 3:** Accuracy of MRT for every sport type, axis and angle.

Sport type	OH		CH		CL			
Angle	Axis							
	45°	90°	45°	90°	45°	90°	Total	Comparison results
Horizontal (Ho)	0.99 ± 0.02	0.93 ± 0.08	0.99 ± 0.01	0.98 ± 0.01	0.99 ± 0.02	0.94 ± 0.07	0.97 ± 0.04	CH90>OH90^b^ CH90>CL90^b^ CL45>CL90^a^ OH45>OH90^a^
Vertical (Ve)	0.99 ± 0.02	0.93 ± 0.07	0.97 ± 0.0	0.95 ± 0.03	0.99 ± 0.01	0.93 ± 0.06	0.96 ± 0.05	CL45>CL90^a^
Longitudinal (Lo)	0.98 ± 0.02	0.98 ± 0.02	0.99 ± 0.02	0.99 ± 0.02	0.98 ± 0.02	0.98 ± 0.01	0.98 ± 0.01	
Total	0.97 ± 0.04		0.98 ± 0.02		0.97 ± 0.03			
Comparison results		L > H^a^		H > V^c^ L > V^b^		L > H^a^ L > V^a^	L > H^c^ L > V^a^	

a p < 0.001.

b p < 0.01.

c p < 0.05.

### 3.2 fNIRS results

#### 3.2.1 Left prefrontal lobe

The fNIRS results showed significant main effects of sport type, F (2, 242) = 6.698, p = 0.001, η^2^ = 0.052, channel, F (5, 238) = 2.513, p = 0.031, η^2^ = 0.050, as well as their interaction, F (10, 478) = 2.780, p = 0.002, η^2^ = 0.055. Other interactions were not significant. Post hoc test analysis revealed that the brain activity of CH group was higher than that of OH and CL group at ch1 (p < 0.001) and ch6 (p = 0.015), as shown in Table 4.

**Table 4:** Oxy-Hb levels of channels in the left prefrontal lobe for every group (β value)/10^−3^.

Channel	OH group	CH group	CL group
ch1	−0.24 ± 1.38	0.63 ± 1.24	−0.49 ± 2.25
ch6	−0.23 ± 1.73	0.40 ± 1.27	−0.02 ± 0.99

#### 3.2.2 Right prefrontal lobe

**Fig. 4:**
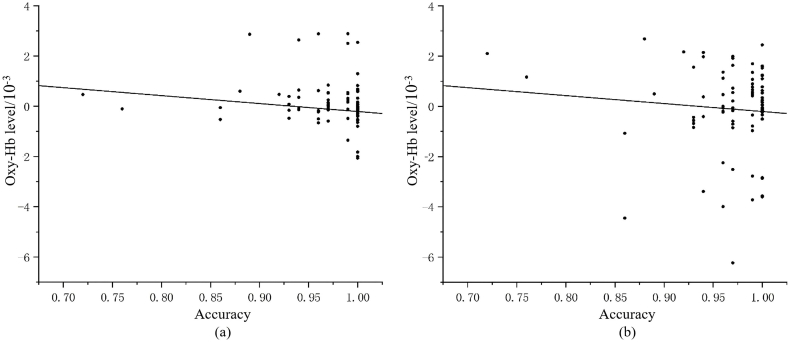
Reaction time of each group performing the mental rotation task.

The fNIRS results showed that the main effect of channel was not significant, F (6, 244) = 1.780, p = 0.104, η^2^ = 0.042. The interaction of channel and sport type was significant, F (12, 490) = 3.108, p < 0.001, η^2^ = 0.071. Further post hoc test analysis revealed significant differences between CH and CL group in brain activity at ch15 (p < 0.001), ch17 (p < 0.001), ch19 (p = 0.026) and ch20 (p = 0.03), as well as the difference between CH and OH group in brain activity at ch15 (p = 0.006), ch17 (p = 0.003), ch19 (p = 0.043), and ch21 (p = 0.012, Table 5). Correlation analysis revealed that the MR accuracy of OH group was correlated with their brain activity at ch15 (r = 0.407, p = 0.038) and ch17 (r = −0.363, p = 0.012, see Fig. 4).

**Table 5:** Oxy-Hb levels of channels in the right prefrontal lobe for every group (β value)/10^−3^.

Channel	OH group	CH group	CL group
ch15	0.05 ± 1.02	−0.54 ± 1.79	0.88 ± 3.15
ch17	−0.21 ± 1.94	0.79 ± 2.54	−0.53 ± 1.70
ch19	0.05 ± 1.01	−0.43 ± 2.02	0.10 ± 0.97
ch20	0.01 ± 1.24	−0.03 ± 0.42	0.20 ± 0.93
ch21	0.14 ± 1.25	−0.50 ± 2.07	−0.07 ± 0.36

#### 3.2.3 Left parietal lobe

**Fig. 5:**
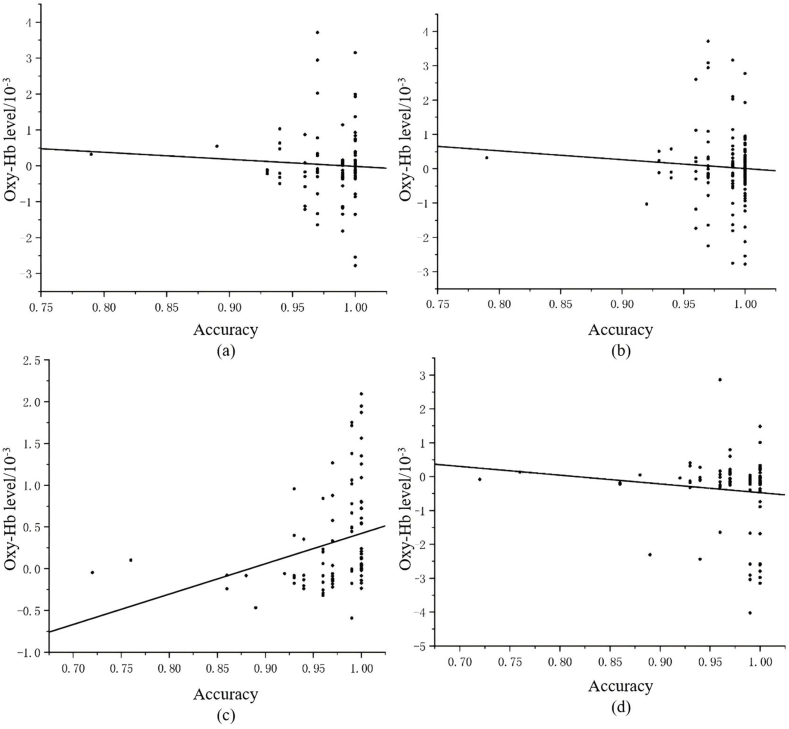
Accuracy of each group performing the mental rotation task.

The results showed significant main effects for channel, F (16, 210) = 5.300, p < 0.001, η^2^ = 0.288, and sport type, F (2, 255) = 9.569, p < 0.001, η^2^ = 0.078. The interaction of channel and sport type was significant, F (32, 422) = 5.343, p < 0.001, η^2^ = 0.078. Post hoc test analysis found different brain activity between the CH and CL group at ch9 (p = 0.003), ch11 (p = 0.038), ch12 (p = 0.012), ch13 (p = 0.009), ch34 (p = 0.005) and ch37 (p = 0.024). also, CH group and OH group differed significantly in brain activity at ch9 (p < 0.001), ch11 (p = 0.023), ch12 (p < 0.001), ch13 (p < 0.001), ch34 (p = 0.038) and ch37 (p < 0.001), and the OH and CL group showed differed brain activity at ch9 (p = 0.002), ch13 (p = 0.001) and ch37 (p < 0.001). The specific results are shown in Table 6. Correlation analysis revealed that the MR accuracy of the CH group was correlated with their brain activity at ch34 (r = −0.238, p = 0.017), and the accuracy of OH group was correlated with the activity at ch9 (r = 0.338, p = 0.001) and ch37 (r = −0.366, p = 0.001, Fig. 5).

**Table 6:** Oxy-Hb levels of channels in the left parietal lobe for every group (β value)/10^−3^.

Channel	CH group	CL group	OH group
ch9	−1.46 ± 4.22	−0.06 ± 1.07	0.35 ± 0.64
ch11	−0.34 ± 1.23	0.04 ± 1.17	0.16 ± 1.67
ch12	−0.29 ± 0.62	−0.11 ± 0.24	−0.03 ± 0.34
ch13	−0.26 ± 0.65	−0.06 ± 0.31	0.10 ± 0.33
ch34	−0.23 ± 0.89	0.19 ± 1.10	0.05 ± 0.94
ch37	0.30 ± 0.96	0.05 ± 0.36	−0.40 ± 1.07

#### 3.2.4 Right parietal lobe

The results showed significant main effects for channel, F (15, 193) = 4.535, p < 0.001, η^2^ = 0.261, and the interaction of channel and sport type, F (30, 388) = 4.717, p < 0.001, η^2^ = 0.267. Post hoc test demonstrated significant differences between CH and CL group in brain activity at ch26 (p < 0.001), ch40 (p = 0.001) and ch44 (p < 0.001). Significant differences were also found between CH and OH group at ch26 (p < 0.001), ch40 (p = 0.028) and ch44 (p < 0.001, Table 7).

**Table 7:** Oxy-Hb levels of channels in the right parietal lobe for every group (β value)/10^−3^.

Channel	CH group	CL group	OH group
ch26	−0.36 ± 0.56	0.11 ± 0.50	0.02 ± 0.30
ch40	0.34 ± 1.14	−0.24 ± 1.17	−0.02 ± 1.05
ch44	−0.68 ± 1.30	0.09 ± 0.73	0.14 ± 0.80

#### 3.2.5 Occipital lobe

The results showed significant main effects for channel, F (4, 231) = 3.960, p = 0.004, η^2^ = 0.064, and sport type, F (2, 234) = 10.023, p < 0.001, η^2^ = 0.079, as well as their interaction, F (8, 464) = 5.006, p < 0.001, η^2^ = 0.079. Post hoc test showed significant differences between the CH and CL group in brain activity at ch46 (p < 0.001) and ch51 (p < 0.001) and differences between CH and OH group in brain activity at ch46 (p < 0.001) and ch51 (p < 0.001, Table 8). Correlation analysis found that the MR accuracy of OH group was correlated with the brain activity at ch46 (r = 0.410, p = 0.003).

**Table 8:** Oxy-Hb levels of channels in the right occipital lobe for every group (β value)/10^−3^.

Channel	OH group	CH group	CL group
ch46	0.22 ± 0.89	−1.25 ± 3.11	−0.06 ± 1.08
ch51	0.02 ± 0.30	−0.34 ± 0.53	0.11 ± 0.50

**Fig. 6:**
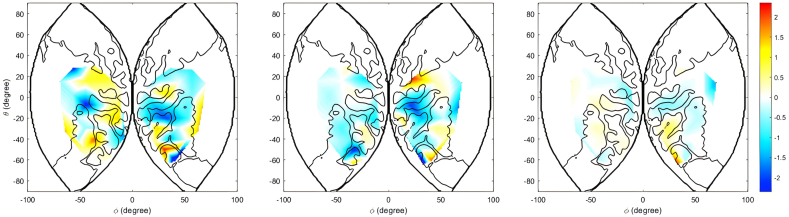
(a) and (b) show the relationship between the activities recorded in ch15 and ch17 and the accuracy of the mental rotation task, respectively. Note: Left for CH athletes, middle for CL athletes, right for ball players.

In addition, this heatmap was placed on the 3D brain model through the Xjview toolbox in MATLAB software (Fig. 6).

## 4 Discussion

### 4.1 Behavioral characteristics of the mental rotation ability of adolescent group

A three-dimensional MRT with different rotation angles and axes was used to test the mental rotation ability of adolescent athletes in different sport types. The behavioral results showed that the performance of small-angle MRT was significantly better than in that of large-angle MRT for the OH and CL group, which is consistent with related studies [37] and supported the hypothesis 1. Consistently, Bethell-Fox et al. concluded that the more difficulty of the stimulus material, the longer it takes the participants to react [38]. Concerning non-significant effect of angle in the CH group, the embodied cognition suggests that an individual's environmental perception could affect one's cognitive ability [15]. In the present study, the CH group (i.e., free skiing players) could adopt more body-related experience learned from the first-person perspective to accelerate the judgements of postures, thus showed similar performance between small and large angle.

The behavioral results for the difference of sport type have demonstrated some significant findings. Firstly, the performance advantage for CH group was showed for large angle for every rotation axes, compared with the OH and CL group. Action imitation theory states that the motor representations and action execution share common processes [19]. Studies have shown that gymnastics and orienteering players have better mental rotation abilities than soccer and running participants do [13,17]. Fargier et al. compared the MR performance of futsal and rhythmic gymnasts and sedentary people and reported that the reaction time of the football group was shorter in the frontal, horizontal, and sagittal planes than that of the sedentary group, and their reaction time in the horizontal plane was also shorter than that of the rhythmic gymnastics [27]. Described as a “circus on the snow”, the freestyle skiing competition is an artistic event comprising twists, backflips and gravity-defying leaps. Therefore, the freestyle skiing sport promoted the development of athletes' mental rotation ability due to the abundant movements of complex multi-axis rotation. The results are partially supported Hypothesis 2. Surprisingly, the OH group showed longer RT than the CL group in 90° vertical rotation, which is inconsistent with our hypothesis and previous studies [39]. It is possible that the defensive movements in basketball and soccer generally require the body to face the opponent (i.e., the brain is more familiar with processing the front of the player's body rather than the side), hence the speed may be slower than the CL group when judging images of the body's side (90° vertical rotation).

In respect to the effect of axis on athletes’ mental rotation, the overall result revealed that the longitudinal rotation had much better performance than the horizonal and the vertical rotation. In terms of the rotation axis, most previous studies carried out paper-plane rotation (i.e., the longitudinal axis in the present study) [2,40], which make this kind of rotation faster and more accurate.

### 4.2 Brain activation characteristics of mental rotation ability in adolescent athletes

The behavioral data such as RT and accuracy found that rotation angle, rotation axis, and sport expertise lead to the difference in mental rotation ability. However, behavioral data cannot reflect the brain cognitive resource allocation during mental rotation. Hence, fNIRS monitoring of frontal, parietal, and occipital lobe in the brain areas of adolescent athletes was used to explore the relationship between different sport types and brain activity of mental rotation tasks in athletes of and their cognitive characteristics.

In the left and right frontal regions, group in the CL and OH groups had significantly greater brain area activation in the right dorsolateral superior frontal gyrus (ch15), right middle frontal gyrus (ch19), and right precentral gyrus (channels 20 and 21) than the CH group did, whereas the CH group had significantly greater brain area activation in the left dorsolateral superior frontal gyrus (ch1), left precentral gyrus (ch6), and right precentral frontal gyrus (ch17). First, in terms of sports characteristics, complicated rotational elements in freestyle skiing allow players to simplify their spatial coding strategies during mental rotation tasks, thereby reducing the brain resources. The higher level of frontal left channel activation in the CH group than in the CL and OH group maybe because the left frontal area is associated with the control and simulation of movement [41]. The present mental rotation task may facilitate the occurrence of motion simulation in athletes, allowing CH group to produce greater activation in ch1 and ch6. Second, in a visuospatial rotation experiment, Suchan et al. reported that the activation focus of the rotation effect was in the right hemisphere of the frontal lobe [42]. The data from the present study are generally consistent with these studies and our hypothesis 3. However, the ch17 results are inconsistent with the above findings, possibly because the greater number of cognitive resources consumed by the CH group enabled them to increase cognitive efficiency to make more capable of mental rotation than the athletes in the CL and OH group. Positive correlation between blood oxygenation (as recorded in ch17) and the accuracy of mental rotation task in ball group was also confirmed.

The results for the parietal lobe showed that ch9 and ch13 belong to the postcentral gyrus, that the CH group had significantly lower activation than the CL and OH groups, and the OH group athletes had significantly lower activation than the CL group, suggesting that CL group require more nutrients in these channels during mental rotation tasks. Zhang et al. found that people at higher altitudes had lower mental rotation scores and reduced gray matter volume in the postcentral gyrus [43]. This finding is also consistent with our behavioral data in which ball players showed a positive correlation between blood oxygen and accuracy in ch9. Ch40 is in the superior parietal gyrus and Ch26 is in the right superior limbic gyrus. These activation levels in brain regions were consistent with Booth's study, which revealed that healthy adults showed more activation in the superior parietal gyrus and surrounding areas when performing a mental rotation task than did pediatric patients and less activation in the supramarginal gyrus [44]. The superior parietal gyrus and postcentral gyrus belong to the superior parietal lobule and that the marginal parietal gyrus belongs to the inferior parietal lobule, which could be related to the mechanism of parietal involvement in mental rotation tasks in athletes with different levels of mental rotation abilities [45]. Because the parietal cortex is associated with spatial process in a egocentric transformation [46], Jagaroo et al. suggested that parietal activation may act as a spatial coordinate transformation, which is related to mental rotation ability [47]. Li et al. found that the left precuneus was associated with reduced P300, that sleep deprivation enhanced effective connectivity in the left inferior parietal lobule and left precuneus, and that mental rotation capacity was negatively associated with effective connectivity [48]. This finding is also consistent with behavioral data, where the performance of CH group were negatively correlated with brain activity at ch34. The CH group had higher activation levels at ch37 (left angular gyrus) than the other two groups, as well as greater activation in the CL group than in the OH group. Halari et al. reported activation in the left angular gyrus in subjects with greater mental rotation capacity [49]. This part of ch44 (right angular gyrus) activity may be related to the spatial transformation. In fMRI studies, this part of the brain is activated when subjects make visual-spatial judgments, such as length or angle comparisons [50]. The lower brain activation in the CH group may because they consumed resources more efficiently than the other two groups during the performance of the mental rotation task, which is consistent with the study of Folkierska-Zukowska et al., who found that subjects with high mental rotation scores had lower levels of activation in the right angular gyrus [51]. In addition, the CH group demonstrated lower activation levels in brain areas corresponding to ch11 (left inferior parietal limbic gyrus) and ch12 (left supramarginal gyrus), and the inferior parietal limbic gyrus, supramarginal gyrus, and angular gyrus were responsible for executive functions, emotional processing, and language integration [52]. Therefore, CH group are more efficient in these channels, i.e., they consume fewer resources and have deeper cognitive processing.

In the occipital lobe region, significant differences were found between activity in ch46 and ch51 (in the supraoccipital gyrus). The supraoccipital gyrus is known to be involved in visual perception, visual mental imagery, visuospatial working memory, and spatial attention. Therefore, differences in the supraoccipital gyrus region may indicate that CL and OH group need more cognitive resources in mental rotation tasks to reach the level achieved by CH group.

### 4.3 Conclusion

The present study investigated the spatial ability of adolescent athletes with different sport expertise of axial rotation, providing the cognitive characteristics from both behavior and fNIRS tests. Since freestyle skiing contains rich spatial elements of the sport, the CH group showed better performance than the OH and CL group for large angle for every rotation axes. The fNIRS results showed the CH group had greater brain activation than the OH and CL group in the left dorsolateral superior frontal gyrus, precentral gyrus, postcentral gyrus, superior parietal gyrus, left angular gyrus, left precuneus and middle occipital gyrus. The findings provide additional empirical support for relating body rotation experience to spatial cognition performance.

## CRediT authorship contribution statement

**Tian Feng:** Methodology, Funding acquisition, Conceptualization. **Fuchun Zhang:** Formal analysis, Data curation. **Manqi Liang:** Software, Resources. **Jinzhao Liu:** Formal analysis, Data curation. **Youxin Wei:** Data curation.

## Informed consent statement

Informed consent was obtained from all subjects involved in the study.

## Ethics approval statement

This study was carried out ethically and approved by the Ethical Committee of Henan Sport University (No.2022002).

## Data and code availability statement

The raw data file “data.xlsx” have been deposited at OSFHOME (https://osf.io/ywz37) with accession numbers 10.17605/OSF.IO/CVJF6.

## Funding information

This work was supported by the 10.13039/501100012456National Social Science Fund of China, grants No. 19CTY013 awarded to Tian Feng (15-07-2019), 10.13039/501100018607Henan Province Science and Technology Project, grants No. 222102320157 to Tian Feng (01-12-2022) and Postdoctoral Research Funding of Henan Province, grants No. 348764 awarded to Tian Feng.

## Declaration of competing interest

All authors declare no conflict of interest.
